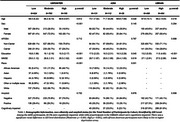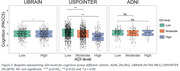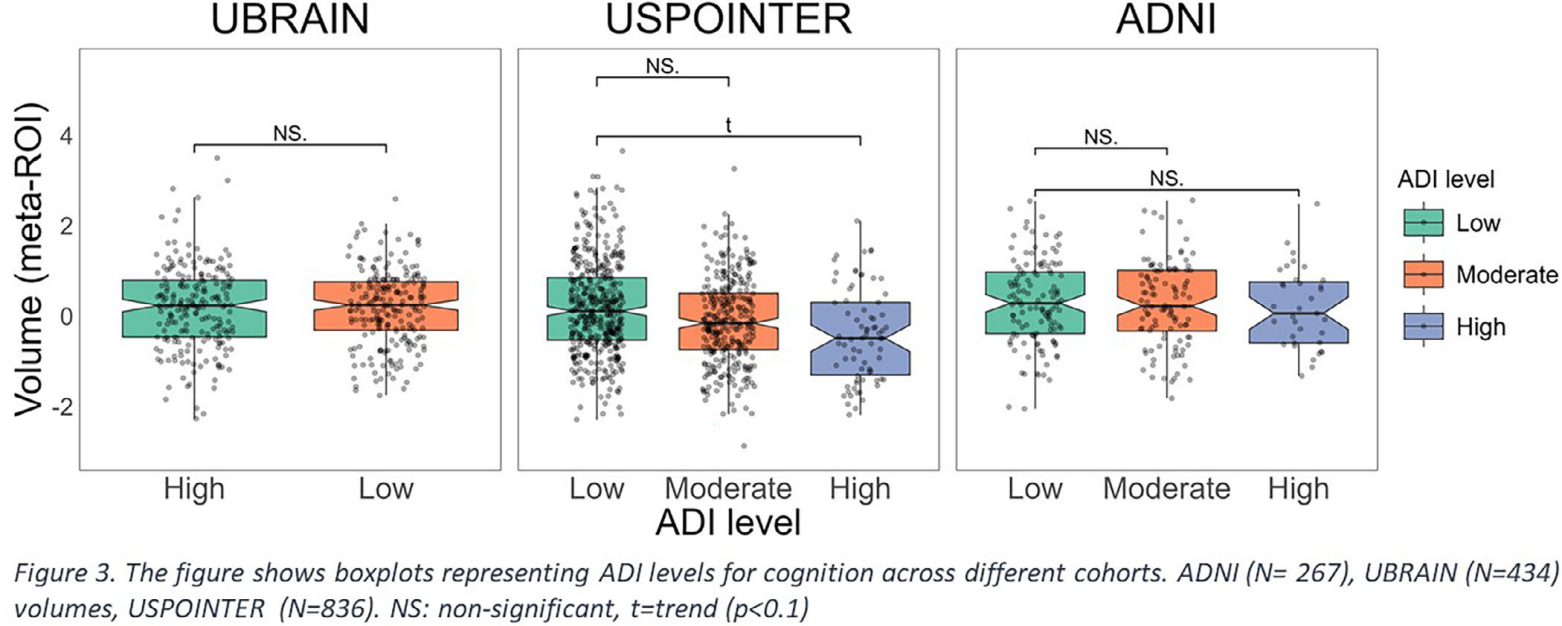# Sociodemographic associations with AD biomarkers in POINTER, UBRAIN, and ADNI

**DOI:** 10.1002/alz70860_101102

**Published:** 2025-12-23

**Authors:** Pablo Aguilar, Carmen M Colceriu, Alex López, María Franquesa‐Mullerat, Sara E Zsadanyi, Agnès Pérez‐Millan, Sarah Tomaszewski Farias, Adrià Tort‐Merino, Thomas Monroe Holland, Sam N. Lockhart, Joseph C. Masdeu, Lycia Tramujas Vasconcellos Neumann, Antonia Valentín, Sami Petricola, Juan Fortea, Alberto Lleó, Heather M Snyder, Laura D Baker, Raquel Sánchez‐Valle, Alexandre Bejanin, Susan M. Landau, Eider M Arenaza‐Urquijo

**Affiliations:** ^1^ Sant Pau Memory Unit, Department of Neurology, Hospital de la Santa Creu i Sant Pau, Barcelona, Spain; ^2^ Global Health Institute Barcelona (ISGlobal), Barcelona, Spain; ^3^ University of Pompeu Fabra (UPF), Barcelona, Spain; ^4^ Sant Pau Memory Unit, Department of Neurology, Hospital de la Santa Creu i Sant Pau, Institut d'Investigació Biomèdica Sant Pau (IIB SANT PAU), Facultad de Medicina ‐ Universitat Autònoma de Barcelona, Barcelona, Spain; ^5^ Hospital de la Santa Creu i Sant Pau, Biomedical Research Institute Sant Pau, Barcelona, Spain; ^6^ Sant Pau Memory Unit, Hospital de la Santa Creu i Sant Pau, Biomedical Research Institute Sant Pau, Universitat Autònoma de Barcelona, Barcelona, Spain; ^7^ Center of Biomedical Investigation Network for Neurodegenerative Diseases (CIBERNED), Madrid, Spain; ^8^ Universitat Autonoma de Barcelona, Barcelona, Spain; ^9^ Alzheimer's disease and other cognitive disorders Unit. Hospital Clínic de Barcelona. Fundació de Recerca Clínic Barcelona – IDIBAPS. University of Barcelona, Barcelona, Spain; ^10^ Alzheimer's disease and other cognitive disorders unit, Hospital Clínic, IDIBAPS, Barcelona, Spain; ^11^ Institute of Neurosciences. Department of Biomedicine, Faculty of Medicine, University of Barcelona, Barcelona, Spain; ^12^ eHealth Center, Faculty of Computer Science, Multimedia and Telecommunications, Universitat Oberta de Catalunya, Barcelona, Spain; ^13^ Alzheimer's disease and other cognitive disorders Group. Service of Neurology, Hospital Clínic de Barcelona. Fundació Recerca Clínic Barcelona‐IDIBAPS, Barcelona, Spain; ^14^ Centro de Investigación Biomédica en Red en Enfermedades Neurodegenerativas (CIBERNED), Madrid, Spain; ^15^ University of California, Davis, Sacramento, CA, USA; ^16^ University of California, Davis School of Medicine, Sacramento, CA, USA; ^17^ Alzheimer's Disease and Other Cognitive Disorders Unit, Neurology Department, Hospital Clinic, Barcelona, Spain; ^18^ Alzheimer's disease and other cognitive disorders Unit. Hospital Clínic de Barcelona; FRCB‐IDIBAPS; University of Barcelona, Barcelona, Spain; ^19^ Hospital Clínic de Barcelona ‐ Fundació de Recerca Clínic Barcelona – IDIBAPS ‐ University of Barcelona, Barcelona, Catalonia, Spain; ^20^ Rush Institute for Healthy Aging, Chicago, IL, USA; ^21^ Rush University Medical Center, Chicago, IL, USA; ^22^ Wake Forest University School of Medicine, Winston‐Salem, NC, USA; ^23^ Wake Forest School of Medicine, Winston‐Salem, NC, USA; ^24^ Houston Methodist Research Institute, Houston, TX, USA; ^25^ Alzheimer's Association, Chicago, IL, USA; ^26^ Department of Neurology, Institut d'Investigacions Biomèdiques Sant Pau ‐ Hospital de Sant Pau, Universitat Autònoma de Barcelona, Hospital de la Santa Creu i Sant Pau, Barcelona, Spain; ^27^ Hospital de la Santa Creu i Sant Pau, Barcelona, Barcelona, Spain; ^28^ Sant Pau Memory Unit, Hospital de la Santa Creu i Sant Pau, Institut de Recerca Sant Pau ‐ Universitat Autònoma de Barcelona, Barcelona, Spain; ^29^ Center for Biomedical Investigation Network for Neurodegenerative Diseases (CIBERNED), Madrid, Madrid, Spain; ^30^ CIBERNED, Network Center for Biomedical Research in Neurodegenerative Diseases, National Institute of Health Carlos III, Madrid, Spain; ^31^ Wake Forest University, Winston‐Salem, NC, USA; ^32^ Wake Forest University Health Sciences, Winston Salem, NC, USA; ^33^ Hospital Clínic de Barcelona, Barcelona, Spain; ^34^ Neuroscience Department, University of California, Berkeley, Berkeley, CA, USA; ^35^ Mayo Clinic, Rochester, MN, USA; ^36^ ISGlobal ‐ Barcelona Institute for Global Health, Barcelona, Catalunya/Barcelona, Spain

## Abstract

**Background:**

In aging and dementia research, the relationships between modifiable risk factors and biomarkers often vary across cohorts. We aim to evaluate the consistency and generalizability of the association between the area deprivation index (ADI) ‐ an indicator of socioeconomic/resource disadvantage within a specific area ‐ and cognition and Alzheimer's disease (AD) biomarkers across three distinct research cohorts, two from the U.S. (U.S. POINTER Imaging, ADNI) and one from Spain (UBRAIN).

**Method:**

We included a total of 3091 participants without dementia, 1879 from U.S. POINTER Imaging, 282 from ADNI and 930 from UBRAIN (Table 1). The ADI was calculated using validated indices from the US and Spain, with neighborhood deprivation ranked as Low, Moderate, and High in the US, and Low and High in Spain. Outcomes of interest included (1) the preclinical Alzheimer's cognitive composite (PACC‐5), (2) MRI‐derived gray matter volumes in AD‐vulnerable regions and (3) PET or CSF‐derived amyloid status (+/‐). We ran logistic regression models with ADI level as predictor adjusted by sex, age, and APOE4 and considered education and race or ethnicity (only US cohorts) in our analyses.

**Result:**

Participants in more deprived areas had lower education across cohorts and were more likely to be female (U.S. POINTER) and African American (U.S. POINTER and ADNI, Table 1). Independent of age, sex and APOE4 status, higher ADI was associated with lower PACC in U.S. POINTER (Moderate: β=‐0.165, *p* <0.001; High β=‐0.412, *p* <0.001) and UBRAIN (ADI: High β=‐0.096, *p* = 0.022) but not in ADNI (Figure 1). After controlling for education, this association remained significant in U.S. POINTER (Moderate: β=‐0.166, *p* = 0.002; High: β=‐0.248, *p* = 0.007) but not in UBRAIN (β=‐0.021, *p* = 0.66). In U.S. POINTER, there was a trend towards an association of higher ADI with lower GM volume in AD‐vulnerable regions (β=‐0.126, *p* = 0.089) that was not observed in ADNI or UBRAIN (Figure 2). No associations between ADI and amyloid status were found across cohorts.

**Conclusion:**

We observed differing associations in neighborhood deprivation and cognition across US cohorts, as well as between the US and Spain cohorts. Country‐specific factors and sample representativeness must be considered when investigating the pathophysiologic mechanisms underlying social determinants and disparities in AD.